# Optimizing Tissue Sampling for the Diagnosis, Subtyping, and Molecular Analysis of Lung Cancer

**DOI:** 10.3389/fonc.2014.00253

**Published:** 2014-09-22

**Authors:** Linda Marie Ofiara, Asma Navasakulpong, Stephane Beaudoin, Anne Valerie Gonzalez

**Affiliations:** ^1^Respiratory Medicine Division, Department of Medicine, McGill University Health Centre, Montreal Chest Institute, Montreal, QC, Canada; ^2^Pulmonary and Respiratory Critical Care Division, Faculty of Medicine, Prince of Songkla University, Hatyai, Thailand

**Keywords:** lung cancer, diagnosis, ultrasound bronchoscopy, diagnostic yield, transthoracic needle aspiration, molecular biomarkers, EGFR

## Abstract

Lung cancer has entered the era of personalized therapy with histologic subclassification and the presence of molecular biomarkers becoming increasingly important in therapeutic algorithms. At the same time, biopsy specimens are becoming increasingly smaller as diagnostic algorithms seek to establish diagnosis and stage with the least invasive techniques. Here, we review techniques used in the diagnosis of lung cancer including bronchoscopy, ultrasound-guided bronchoscopy, transthoracic needle biopsy, and thoracoscopy. In addition to discussing indications and complications, we focus our discussion on diagnostic yields and the feasibility of testing for molecular biomarkers such as epidermal growth factor receptor and anaplastic lymphoma kinase, emphasizing the importance of a sufficient tumor biopsy.

## Introduction

Lung cancer remains the leading cause of cancer death in North America. In Canada, an estimated 25,500 Canadians will be diagnosed with lung cancer in 2014 (1). The majority of these will be non-small cell lung cancer (NSCLC) and unresectable.

At diagnosis, 75% of lung cancer patients will have either locally advanced or metastatic disease (2). The goal in this group of patients is to establish the diagnosis and, ideally, confirm staging with the least invasive technique possible. As a result of this approach, biopsy specimens are becoming increasingly smaller. Up to 80% of patients receiving chemotherapy for advanced disease will have only a small biopsy and/or cytology samples available for diagnosis (3).

The adequacy of these samples has important ramifications. Lung cancer has entered an era of personalized therapy with treatment based on histologic subtypes (adenocarcinoma versus squamous) and the presence of molecular markers [epidermal growth factor receptor (EGFR) and anaplastic lymphoma kinase (ALK)]. For instance, several trials have demonstrated that response rate and overall survival is significantly better with pemetrexed in patients with non-squamous histology compared with patients with squamous histology (4). Trials using tyrosine kinase inhibitors (TKIs) have observed that patients with NSCLC tumors harboring EGFR mutations derive a greater benefit from treatment with TKIs than wild-type tumors (5). In fact, a number of trials have consistently shown a statistically significant and clinically meaningful benefit of TKIs over standard chemotherapy in mutation positive patients (5–7). The ALK inhibitor, crizotinib, is effective in patients with NSCLC harboring the ALK rearrangement (8).

Procurement of adequate tissue samples that allow for accurate characterization of histology and molecular testing is essential. A multidisciplinary approach is recommended. Physicians who obtain tissue samples (respirologists, interventional radiologists, and thoracic surgeons) need to be aware of the tissue yields of their procedures. Likewise, pathologists need to communicate the tissue yields and to be judicious in tissue use especially when managing small biopsy and cytology specimens. Finally, medical oncologists should be aware of when to ask for more tissue in patients in whom the treatment plan will be significantly impacted by further characterization. Medical oncologist may recommend that a patient with a known lung cancer be rebiopsied or that a metastatic site be biopsied in addition to the primary site in order to clarify the molecular status of the tumor. This can provide important information with regard to treatment options or as to why therapies fail.

In this article, techniques used in the diagnosis of lung cancer will be discussed including the expected tissue yields and the feasibility of histologic characterization and molecular testing.

## Diagnosis of Lung Cancer

### Rapid assessment clinics

Lung cancer guidelines recommend prompt investigation and referral for treatment (9).

Recently, rapid access clinics have been developed to reduce wait times and initiate investigations based on established algorithms to provide the most information about diagnosis and staging with the least risk to the patient. Bronchoscopy with or without lymph node sampling is frequently recommended as the initial diagnostic procedure.

### Fiberoptic bronchoscopy

The bronchoscope is one of the primary diagnostic tools in lung cancer. Flexible bronchoscopy, usually performed under local anesthesia and with minimal sedation, provides a thorough examination of all segmental bronchi within minutes. Complications for this procedure are rare, with major complication rates between 0.08 and 5% (10). Complications include pneumothorax, hypoxemia, and hemorrhage (11).

**Figure 1:**
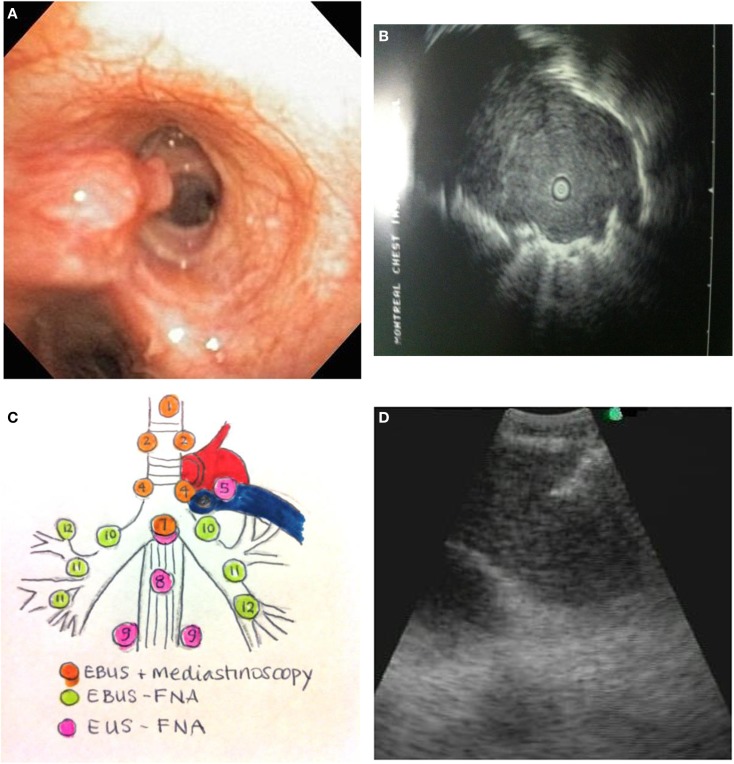
**(A)** Endobronchial tumor visible in an airway. **(B)** Ultrasound image of a peripheral lung cancer as visualized by radial EBUS-GS. The clear central area is the ultrasound probe in the airway. The surrounding isoechoic shadow represents a tumor. The hyperechoic line surrounding the tumor is an ultrasound phenomenon produced by the sudden change in tissue density from tumor to aerated lung. **(C)** Mediastinal lymph node station accessibility by EBUS, mediastinoscopy, and EUS. **(D)** Real-time needle aspiration of a lymph node. The needle (hyperechoic line coming from the top left corner of the screen) is penetrating the lymph node under direct ultrasound visualization.

Endobronchial tumor may be visible as an exophytic mass or submucosal infiltration (Figure 1A). The diagnostic yield for endobronchial biopsy when a lesion is visible is 70–90% (12). Five biopsy specimens have been shown to be optimal for achieving a diagnostic yield in central lesions (13). Combining the results of bronchial biopsy, bronchial brushing, and bronchial washing increases tissue yields (14), and it is better to do brushing after biopsy (15).

Biopsy specimens are, in general, small averaging about 300 cells in aggregate. Bronchial lavage yields the least number of malignant cells. In biopsy specimens, the percentage (%) of tumor cells can be relatively low. Coghlin et al. found the mean % of area of tumor in an endobronchial sample to be 33%. In fewer than half of their cases (48%), tumor was found in all biopsy specimens (16). Although five specimens may be enough to establish the diagnosis of lung cancer, the number of specimens required to provide detailed sub classification and molecular analysis has not be established. In one series, EGFR testing could be performed in 100% of endobronchial biopsy specimens that established a diagnosis of lung cancer (17).

Endobronchial cryobiopsies could be one evidence-based way of achieving a higher diagnostic yield and a higher molecular analysis potential. Compared with conventional bronchoscopic biopsies, cryobiopsies result in an increase in biopsy sample size and yield (18, 19).

In the case of more peripheral lesions, when the endobronchial exam is normal, the diagnostic yield falls to 40% (20, 21). The diagnostic yield can be increased when computed tomography (CT) images are available for review prior to bronchoscopy (22). This allows the bronchoscopist to better localize the bronchial segment containing tumor. When positive, the diagnoses in these cases are usually made on the basis of cytology: bronchial brushings or washings. Molecular markers can be performed on these cytological specimens with varying degrees of success. One series, however, found that in the case of bronchial lavage, more than half of the cytological specimens that confirmed the diagnosis of lung cancer could not be used for molecular testing (23).

Ultrasonography using a guide sheath (radial EBUS-GS) and electromagnetic navigation (ENB) can provide transbronchial biopsy specimens, improving the possibility of having adequate tissue for molecular analysis. In the case of a peripheral lesion where the endobronchial exam is negative and radial EBUS-GS or ENB are not available, consideration should be given to other diagnostic procedures such as transthoracic needle aspirate.

### Radial EBUS

Endobronchial ultrasonography using a sheath guide (EBUS-GS) can increase the diagnostic yield of peripheral lung lesions. For lesions less than 2 cm, the diagnostic yield can increase from 36% using conventional bronchoscopy to between 58 and 70% (24). This technique allows for visualization of the lesion (Figure 1B) and repeated access to the lesion by brush, forceps biopsy, and bronchial wash. The resulting specimens are cytological and small biopsies.

Recently, ENB and virtual bronchoscopic navigation system (VBNS) have been developed to assist the diagnosis of peripheral lung lesions in conjunction with EBUS-GS. Using ENB, yields in peripheral lesions can be further improved upon. Combining radial EBUS and ENB resulted, in one series, in a diagnostic yield approaching 90% compared with 69% for radial EBUS alone (25). No EMN complications have been reported. VBNS has also been used with EBUS-GS with an overall diagnostic yield ranging from 63.3 to 84.4%, and in lesions less than 2 cm in diameter, ranging from 44 to 75.9% (26). VBNS increases diagnostic yield and decreases procedure time (27). Presently, there is little data on the yield of molecular testing on specimens obtained by EBUS-GS or ENB/VBNS. Tsai et al. performed EBUS-guided brushings in 122 patients with peripheral lung cancer receiving flexible bronchoscopy. The yield for tumor cells was 68.9%. Genotyping of EGFR and KRAS was successfully implemented in 80 (95.2%) of the 84 cytology-proven brushing samples (28). It is probable that the yields are similar to conventional bronchoscopy as the specimens obtained are small biopsies and bronchial brushing/lavage cytology.

### EBUS transbronchial needle aspiration

Endobronchial ultrasound-guided transbronchial needle aspiration (EBUS-TBNA) is a minimally invasive technique with a high diagnostic yield for mediastinal lymph node staging of lung cancer patients. Accurate staging is an essential step in the investigation of lung cancer patients. EBUS-TBNA is particularly useful as diagnosis and staging can be achieved with a single procedure.

The technique is performed using a dedicated flexible bronchoscope with an integrated ultrasound transducer. It allows for sampling of mediastinal and hilar lymph nodes under direct vision using local anesthesia and moderate sedation. The upper and lower paratracheal, prevascular, subcarinal, and hilar lymph node stations can all be sampled using this technique (Figure 1C).

A similar technique using a gastroscope with an integrated ultrasound probe (EUS) can also sample mediastinal lymph nodes. Nodal stations that can be accessed with EUS include aortopulmonary window, subcarinal, para-esophageal, and pulmonary ligament.

Herth et al. assessed EBUS yields in 502 patients with suspected lung cancer, comparing EBUS-TBNA results with operative findings (29). The reported sensitivity was 94% and specificity was 100%. Several studies have compared EBUS-TBNA to mediastinoscopy and found both techniques to have comparable results for mediastinal staging (30, 31). EBUS-TBNA has some advantages over mediastinoscopy, in that EBUS-TBNA can be used to restage a patient post surgery or radiation therapy, where a repeat mediastinoscopy would prove difficult because of fibrotic changes (32). Additionally, it can be performed in high-risk patients with several comorbidities such as COPD (33).

Tissue samples by EBUS-TBNA are typically small cytology samples obtained using a dedicated 22 gage needle (Figure 1D). Some institutions use rapid on-site evaluation (ROSE) of aspirated samples by a cytopathologist. One of the main advantages of ROSE is reduction of the number of passes and stations sampled, and avoidance of other biopsy techniques like transbronchial biopsy. Lee and colleagues have demonstrated that maximum diagnostic values for achieving a diagnosis of lung cancer are achieved with three aspirations per node when ROSE is not available (34). Molecular testing for EGFR and ALK mutations can be successfully performed on EBUS-TBNA specimens. In several series, using ROSE, molecular testing can be performed in between 70 and 90% of EBUS-TBNA samples (35–37). Yarmus et al. found that a median of four passes in the presence of ROSE provided an adequate amount of tissue for molecular analysis in 95% of patients studied (38). In the absence of ROSE, Navasakulpong and colleagues found that 93% of EBUS-TBNA specimens from a single lymph node station were adequate for EGFR testing with an average of 3.5 passes per lymph node. The minimum tumor cell count that allowed for successful EGFR testing in this series was 100 cells (39). Schmid-Bindert et al. found that EBUS-TBNA provided the highest yield for biomarker testing when compared to bronchoscopic forceps biopsy and CT-guided core biopsy (17).

Questions that remain to be answered are whether a larger needle (21 gage) results in better yields, whether mixing tissue from more than one lymph node station, once staging is established, can improve the yield of molecular testing, and finally, whether combining EBUS and EUS increases tissue yields for molecular analysis.

### Mediastinoscopy

Cervical mediastinoscopy is used predominantly in the staging of lung cancer. It is performed by a thoracic surgeon under general anesthesia in an operating room. A small incision is made at the base of the neck and a mediastinoscope is introduced. The sensitivity of mediastinoscopy for detecting cancer in mediastinal lymph nodes is between 80 and 95% (32, 40). False negative rates vary between 5 and 9% and are attributed to the inability to access para-esophageal, inferior pulmonary ligament, and aortopulmonary nodes.

Tissue samples vary from millimeters to centimeters depending on the size of the nodes biopsied. Tissue samples are sufficient for molecular testing. The complication rate is between 2 and 5% and includes hoarseness, infection, and bleeding (41).

Several series have compared EBUS to mediastinoscopy (42). Both modalities have comparable sensitivities in staging the mediastinum. Mediastinoscopy has the advantage of larger tissue samples, compared with EBUS. It is unclear if this translates into better molecular subtyping as little comparative data exist. The disadvantage of mediastinoscopy is the need for general anesthesia and OR time.

### Transthoracic needle aspirate

A total of 10–20% of cases of NSCLC will present as a solitary pulmonary nodule. In patients who are not candidates for surgery or in patients who have advanced disease in whom the most accessible site for biopsy is a peripheral lung nodule, transthoracic needle aspiration (TTNA) and biopsy (TTNB) are useful diagnostic procedures.

Transthoracic needle aspiration can be performed under CT or fluoroscopic guidance. CT-guided aspiration and biopsies result in a higher diagnostic yield compared to fluoroscopy (43).The most commonly used technique is a coaxial system, in which a larger gage needle is inserted into the edge of the lesion and a smaller needle is passed through the larger one. This allows for a single pleural puncture and repeat needle passes by the smaller needle reducing the risk of complications. Major complications are bleeding and pneumothorax and occur in 10% and up to 20% of cases, respectively (44). Contraindications to TTNA are previous pneumonectomy, severe chronic obstructive lung disease, especially with bullous formation, mechanical ventilation, lesions too close to vascular structures, and high risk for bleeding (45).

Transthoracic needle aspiration has a diagnostic accuracy of between 80 and 95% for lung cancer (46, 47). Specimens obtained by TTNA are cutting-needle core biopsies and needle aspirate cytology. Core-needle biopsy specimens usually contain enough cellular material for pathologic subtyping and molecular analysis. The average number of cells obtained by CT-guided needle biopsy is 500 cells per biopsy (48). Zhuang et al. showed that CT-guided TTNA/TTNB performed using an 18 or 20 gage could obtain tumor samples ranging from 0.5 to 1.5 cm in length and that these samples were 100% adequate for histological and EGFR mutation analysis (49). In addition, Fassina et al. showed that TTNA samples can be used for EGFR and KRAS mutation analysis (50). da Cunha Santos et al. found that in a review of 602 fine needle aspirates, histological subtyping agreement with resected specimens was achieved in 93% of cases (51).

### Pleural fluid analysis and medical thoracoscopy

In rapid diagnostic clinics for the evaluation of suspected lung cancer, diagnostic procedures that allow for simultaneous staging and diagnosis are preferred. In patients with suspected lung cancer presenting with an accessible pleural effusion, thoracentesis is recommended to distinguish between a malignant versus parapneumonic effusion (21). The yield of pleural fluid cytology is 60–80% with repeat sampling (52, 53). Use of cell block methods improves the diagnostic utility of pleural cytology compared with conventional smear cytology by providing higher cellularity and better morphological features to allow for pathologic subtyping. Using cell blocks of pleural fluid, molecular testing for EGFR and KRAS has been performed with an insufficiency rate of 3.7% (1 in 27 specimens) (54).

Medical thoracoscopy is recommended when cytology specimens are non-diagnostic or insufficient for histologic classification. It offers higher yield compared with Abrams needle and CT-guided pleural biopsy in malignant pleural disease (55). In addition to being able to directly visualize and biopsy nodules on the parietal pleural surface, thoracoscopy allows for drainage of pleural fluid and talc pleurodesis in the case of malignant effusions.

Medical thoracoscopy can be performed in a dedicated sterile endoscopy suite under local anesthesia and conscious sedation. A pneumothorax is artificially induced, and a rigid thoracoscope is introduced into the pleural cavity. Under direct vision, parietal pleural nodules can be biopsied. The diagnostic yield of medical thoracoscopy for malignancy is 93–97% (56). Biopsy specimens are typically about 5 mm and multiple specimens can be obtained during the procedure. The size of these specimens is adequate for pathological subtyping, and molecular analysis was possible in 100% of specimens tested in one series (57).

Medical thoracoscopy is a relatively safe procedure with a complication rate of 1.9% (58). Persistent air leak, subcutaneous emphysema, and fever are the most common complications. Mortality is rare with 1 death reported in more than 8000 cases (53).

## Tissue Strategies for Pathological Subtyping and Molecular Analysis

Strategies have been proposed to allow for subtyping of NSCLC and testing of molecular markers in small biopsy and cytology specimens (59). With any specimen, the first approach is to establish squamous or adenocarcinoma differentiation based on morphology under light microscopy. The typical features of adenocarcinoma include glandular differentiation of cell clusters and in individual cells, the presence of basophilic cytoplasm, eccentric nuclei, and a single macronucleolus. Squamous differentiation is characterized by keratinization, intercellular bridges, and keratin pearls in small biopsies. Individual cells may have long cytoplasmic tails, central nuclei, dense chromatin, and poorly developed nucleoli.

In cases of NSCLC that cannot be subtyped based on morphology, immunohistochemistry (IHC) is used. Because of the small amounts of tissue, IHC should be used judiciously. It is recommended to use one adenocarcinoma marker (TTF1) and one squamous marker (p63 or CK 5/7) to attempt to further subtype NSCLC (60).

In the case of adenocarcinoma, molecular markers can then be performed. Currently, EGFR and ALK are performed, but other markers such as ROS1 and KRAS may also be considered. In tumors that cannot be subtyped based on morphology and IHC, a designation of NSCLC not otherwise specified (NOS) is made. Decisions can be made whether additional tissue is warranted; however, recommendations for EGFR testing include specimens designated as NSCLC-NOS (61).

The minimum number of malignant tumor cells required for molecular marker testing has not been well established. In general, larger samples with at least 200–400 malignant cells are preferred (62).

Communication among the multiple physicians involved in the care of patients with lung cancer must take into consideration issues of tissue procurement strategies in order to optimize diagnostic yield and molecular characterization of tumors. Only a multidisciplinary approach can ensure that the needs of the medical oncologist for treatment planning are reflected into judicious tissue procurement, clinical staging, and thoughtful tissue analysis. Moreover, local institutional strategies must be implemented to take into consideration the local availability of different diagnostic modalities and molecular analyses. Solutions regarding issues of cost-effectiveness and quality control must be individualized for each center, and ongoing monitoring is important to ensure that safe and efficient diagnostic services are delivered. This is especially important given that many of the above-mentioned technologies have mostly been studied only in highly specialized centers.

## Summary and Recommendations

A sufficient tumor biopsy is essential in the diagnosis of lung cancer in order to subtype NSCLC and to establish the presence molecular markers. Important therapeutic decisions are made on the basis of these specimens. In this article, we have summarized the various techniques used in the diagnosis of lung cancer and their respective yields in terms of tissue, pathological subtype, and molecular testing. Table 1 summarizes the diagnostic yields of specimens obtained.

**Table 1:** **Yields of various procedures used to diagnose lung cancer**.

Diagnostic modality	Specimen types	Diagnostic yield	Adequacy for biomarker testing
Bronchoscopy	Endobronchial biopsy	70–90% (if lesion visible)	Up to 100% in one series for endobronchial biopsy. Less than 50% in washings
	Brushing cytology	Yields improve when biopsy, brushing, and washing combined	
	Washing cytology		
Radial EBUS-GS	Transbronchial biopsy	58–70% when biopsy, brushing, and washings combined	71% in one series examining bronchial brushing
For peripheral lesions 2 cm or less	Brushing cytology	
	Washing cytology	
EBUS-TBNA	Needle aspirate cytology	Up to 94%	70–95%
Mediastinoscopy	Biopsy	80–95%	Not well established, but likely adequate based on size
CT-guided TTNA	Core-needle biopsy	80–95%	100% in one series
	Needle aspirate	
Thoracentesis	Fluid cytology	60–80%	Insufficiency rate of 3.7% in one series
Medical thoracoscopy	Biopsy	93–97%	100% in one series

A multidisciplinary approach in establishing a diagnosis of lung cancer is strongly recommended to optimize tissue yields and ultimately patient outcomes. In general, the least invasive procedure should be favored and biopsy specimens favored over cytology specimens. There is, however, increasing evidence to suggest that, when handled judiciously, cytology specimens can prove to be sufficient for diagnosis and molecular analysis. Understanding the yields of diagnostic procedures is essential in diagnosing and treating lung cancer in an era of personalized therapy.

## Conflict of Interest Statement

The authors declare that the research was conducted in the absence of any commercial or financial relationships that could be construed as a potential conflict of interest.
